# Environmental Risk Assessment Resulting from Sediment Contamination with Perfluoroalkyl Substances

**DOI:** 10.3390/molecules26010116

**Published:** 2020-12-29

**Authors:** Grażyna Gałęzowska, Justyna Rogowska, Ewa Olkowska, Wojciech Ratajczyk, Lidia Wolska

**Affiliations:** Department of Environmental Toxicology, Faculty of Health Sciences, Medical University of Gdansk, Debowa Str. 23A, 80-204 Gdansk, Poland; grazyna.galezowska@gumed.edu.pl (G.G.); ewa.olkowska@gumed.edu.pl (E.O.); sinclair@gumed.edu.pl (W.R.); lidia.wolska@gumed.edu.pl (L.W.)

**Keywords:** perfluorinated compounds, PFASs, sediments, environmental risk assessment, liquid chromatography

## Abstract

Due to wide use of perfluoroalkyl substances (PFASs) (e.g., in metal-plating, in fire-fighting foam, lubricants) and their resistance to degradation, they occur widely in the environment. The aim of this study was to estimate the environmental risk resulting from the presence of PFASs in the Gulf of Gdansk. Therefore, 17 PFASs concentrations were determined using ultra performance liquid chromatography with tandem mass spectrometry detection (UPLC-MS/MS). Additionally, sediment ecotoxicity was investigated. The results of the chemical analysis were used to asses environmental risk of PFASs. In samples collected around discharge collectors from a wastewater treatment plant and the Vistula mouth, Σ17PFASs values were 0.00403 ÷ 40.6 and 0.509 ÷ 614 ng/g d.w., respectively. In samples collected around discharge collectors, PFHxA, PFPeA, PFHpA, and PFOA were dominating, while at the Vistula River mouth, PFHxS, PFDS, and PFBS were prevalent. For most sediments, no toxic effect was observed in the toxicity tests with *Heterocypris inconguens* and *Aliivibrio ficsheri*. There was no observed correlation between the PFASs level and their ecotoxicity. Generally, the results of environmental risk assessment indicate that the PFASs would not generate high impact on the aquatic life (five water samples have shown medium risk related to PFBS and PFDoA).

## 1. Introduction

Perfluoroalkyl substances (PFASs) comprise a large and growing class of chemicals that include perfluoroalkyl acids (PFAAs) and especially those categorized as perfluoroalkyl carboxylic acids (PFCAs) and perfluoroalkyl sulfonic acids (PFSAs) [1]. These compounds have been used worldwide in a range of industrial and household consumer products such as textile coatings, paper packaging, fire-fighting foams, insecticides, paints, cosmetic formulations, and semiconductors, since the 1950s [2]. Widespread use and extreme resistance to degradation have resulted in the ubiquitous presence of these compounds in the environment [3]. Therefore, they are toxic and persistent, which resulted in PFSAs, their salts, and perfluorooctyl sulfonyl fluoride (POSF) being labeled as persistent organic pollutants (POPs) under the Stockholm Convention [4,5].

PFASs can be released to the environment during production, use, and disposal [6]. A significant source of PFASs in the aquatic environment are wastewater treatment plants (WTPs) [7]. The removal efficiency depends on the different unit processes used in treatment plants and also on the type of PFASs [8]. For example, Lee et al. (2019) have indicated that existing biological treatment processes are able to eliminate some perfluorononanoic acid (PFNA), perfluoro-*n*-pentanoic acid (PFPeA), and perfluorohexane sulfonate (PFHxS), but most of the other compounds could not be removed through biological treatment [9]. Consequently PFASs are transported with treated wastewater to aquatic environmental compartments (e.g., rivers, lakes, seas). Kibambe et al. (2020) in their research suggest that despite the wastewater treatment process, PFASs were still present in the effluent samples in high concentrations, usually exceeding 1 ng/L, which may present a significant threat to the growth and survival of aquatic organisms in the receiving water bodies [8]. An important pathway for the transport of PFASs from soils to seas and oceans are rivers and partly atmospheric pathways [10,11]. A study carried out by Pan and You (2010) suggests that PFSAs may be carried with river water and transported for long distances before they reach the sea and largely scavenged to the sediment in the estuaries due to the change in salinity [12]. One of the most important environmental sinks and reservoirs for PFASs are sediments [13]. At the same time, it should be noted that sorption of PFSAs from seawater to marine sediments can be approx. 10 times higher than that in freshwater [14]. PFASs from sediment can be transferred to aquatic organisms, leading to bioaccumulation and even trophic biomagnification in aquatic food webs [15]. Moreover, PFSAs and PFCAs cause adverse health effects such as immunotoxicity, hepatotoxicity, and developmental toxicity, and can pose a threat especially to benthic organisms [4].

Therefore, monitoring of PFASs concentrations in sediment with their spatial distributions in the different environments and bioaccumulation in living organisms is urgent and crucial [15]. Several perfluorinated contaminants have been identified in Baltic Sea biota, in humans living on the Baltic coast, or abiotic elements of aquatic ecosystems [16,17]. However, there is still a lack of PFASs’ environmental risk assessment (ERA) in sediments and water samples of large-scale reservoirs. The results of ERA can provide valuable data for anthropogenic pollution control and further management of water resources in the area of the Gulf of Gdansk [15].

The aim of this study was to evaluate the environmental risk resulting from the presence of PFASs in marine sediment and water. According to this research goal, the following steps were done:

- determination of 17 PFASs concentration in sediment samples collected in the area of the Gulf of Gdansk;
- assessment of sediment ecotoxicity and investigation of the relation between measured ecotoxicity results and PFASs concentrations;
- environmental risk assessment due to the presence of PFASs in sediments and water samples (estimated values based on partitioning behavior of pollutants between sediment and surface water).

## 2. Results

### 2.1. Toxicity of Sediment Samples

**Figure 1:**
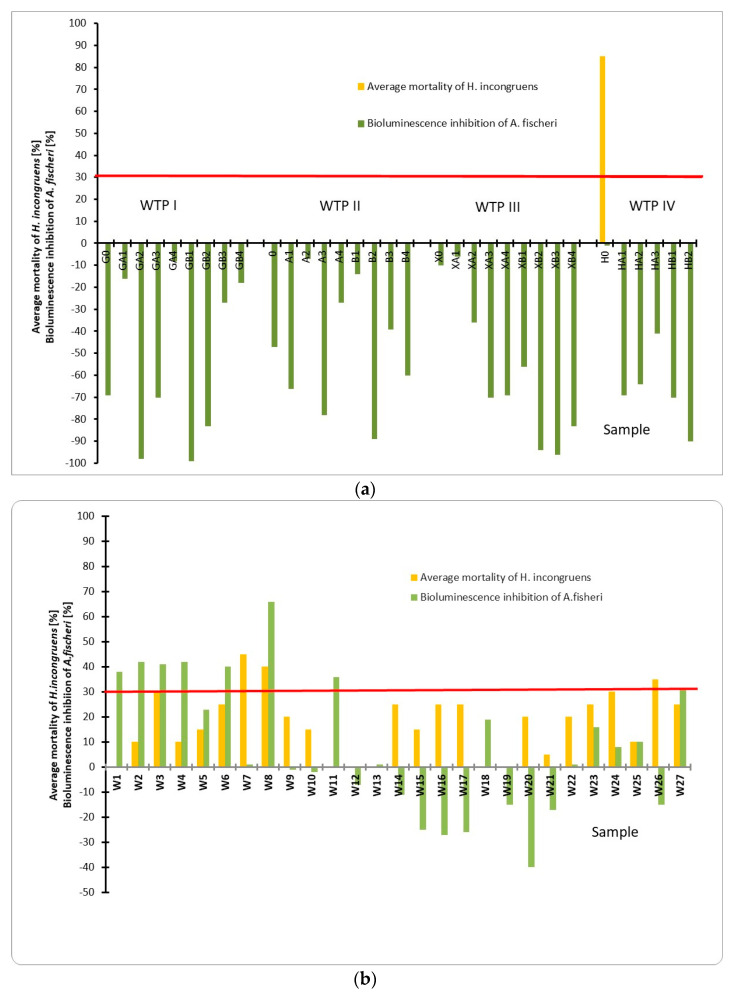
Toxicity of bottom sediments taken around the sewage collectors’ outlets from wastewater treatment plants (WTP I-WTP IV) (**a**) and from the mouth of Vistula (**b**) (samples below the red line have mortality of *H. incongruens* less than 30% and growth inhibition data related to these cases is shown in Figure S1 of the Supplementary Materials).

None of the bottom sediment samples collected around the discharge collectors showed any toxic effect on the *Aliivibrio fischeri* bacteria (Figure 1a). In the case of samples collected at the mouth of the Vistula River, slightly toxic effect on *Aliivibrio fischeri* was observed at points W1–W6, W11, and W27 (38%, 42%, 41%, 42%, 23%, 40%, 36%, and 31%, respectively). Only sample W8 was toxic (66%). (Figure 1b). However, during the tests, a significant increase in the luminescence of bacteria was observed, which indicates the phenomenon of hormesis, a stimulatory response indicator to a compound at low doses.

In samples taken around the collectors, sub-chronic toxicity was determined with the Ostracodtoxkit F test. Toxicity was observed only for one sample located at point H0 (85% mortality) (Figure 1a). In other samples from this area, no toxic effect in the form of mortality was observed. In addition, in the case of samples collected around the IV collector, the growth inhibition effect of the test organism was observed in the samples H0, HA1, HA2, and HA3 at the level of 46%, 41%, 29%, and 22%, respectively (Figure S1). The same effect was observed with sample XA2 collected around collector III. Most of the samples taken around collectors I, II, and III showed a stimulating effect on the tested crustacean. In the case of samples taken at the mouth of the Vistula, toxicity tests on the *Heterocypris incongruens* crustacean showed that the samples W7, W8, and W26 had the highest level of mortality of test organisms (45%, 40%, and 35%, respectively).

### 2.2. Determination of PFASs in Sediment Samples

**Figure 2:**
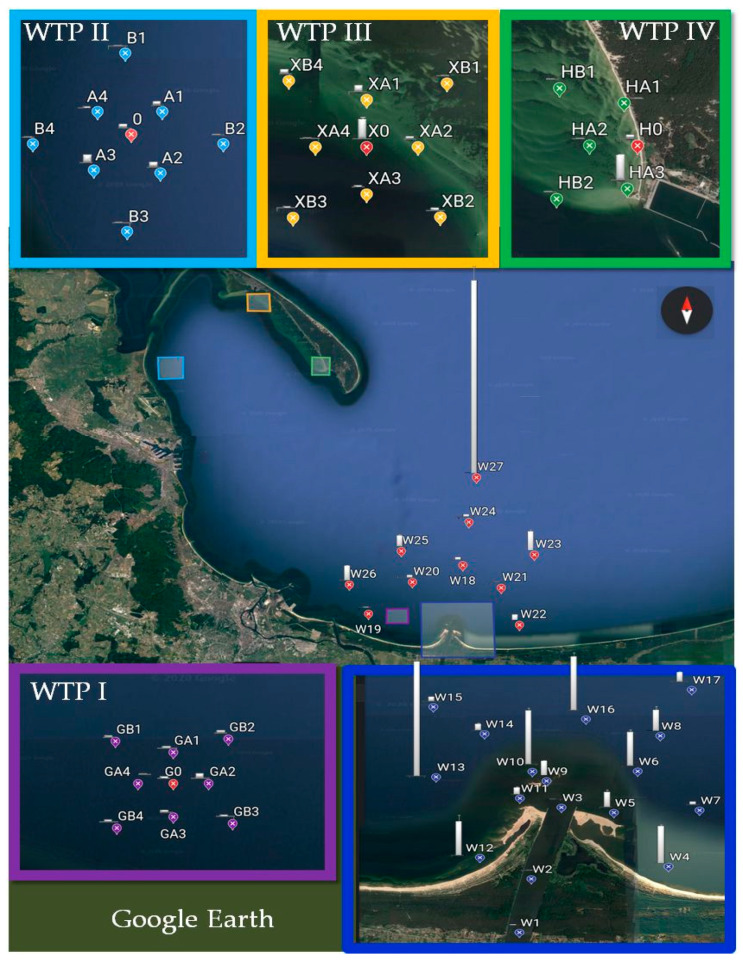
The results of the determination of PFASs in samples taken around the sewage collectors’ outlets from wastewater treatment plants (WTP I-WTP IV) and from the mouth of Vistula.

In the case of samples collected around the discharge collectors, the highest concentrations of ΣPFASs were observed in the HA3 sample (40.6 ± 2.5 ng/g d.w.—WTP IV); X0 (31.5 ± 4.4 ng/g d.w—WTP III) and A3 (12.1 ± 1.2 ng/g d.w—WTP II) (Figure 2, Figure S2). For most of the samples, the concentrations of pollutants did not exceed 4 ng/g d.w. Analyzing the content of individual compounds, it should be noted that PFUdA, PFBA, and PFHpA were determined only in samples collected around the WTP I collector. PFDS was determined only in one sample—GA2. PFBS was determined only in samples collected around the WTP II and WTP III collectors. PFDS was below the detection limit in all samples except sample GA2. The highest concentrations of all determined PFASs were recorded for PFHxA, especially at the HA3 point (WTP IV), A1 (WTP II), and X0 and XA1 (WTP III).

In the case of samples taken at the mouth of the Vistula, the highest concentrations of ΣPFASs were observed for points W13 and W27 (350 ± 20 and 614 ± 46 ng/g d.w., respectively). The content of PFHxS in these samples had influence on the above mentioned results (Figure 2, Figure S2). The lowest level of ΣPFASs was found in the samples collected at W2 (0.509 ± 0.017 ng/g d.w.), W3 (0.79 ± 0.11 ng/g d.w.), and W21 (0.98 ± 0.12 ng/g d.w.). All 17 PFASs were determined only in samples W7 and W6. In almost all samples, PFOS, PFUdA, PFBA, and PFHpA were below the method quantification limit (MQL for individual PFASs was between 0.27 and 11 pg/g d.w.). The highest concentrations, among the various PFASs, have been observed for perfluoroalkyl sulfonic acids, especially in samples W10, W13, W16, and W27.

Furthermore, statistical methods were used to search for the relationship between the obtained results (Statistica, version 13.3; StatSoft—Kraków, Poland).

### 2.3. Environmental Risk Assessment of PFASs

**Figure 3:**
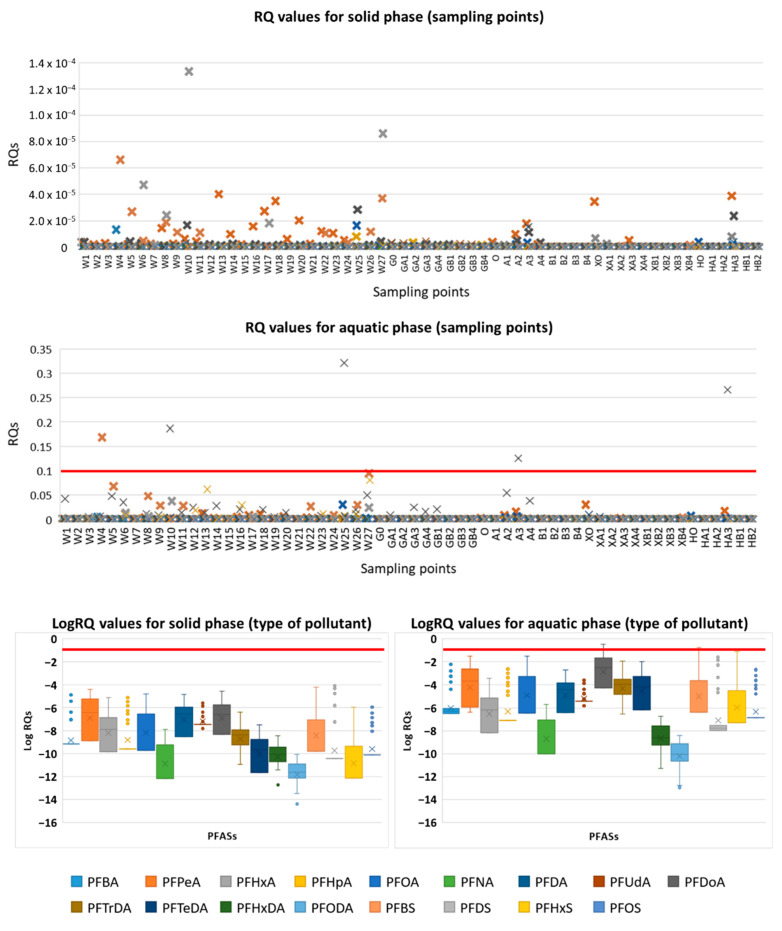
The environmental risk quotients (RQs) of PFASs compounds in the Gulf of Gdansk (the Vistula River: W1–W27; wastewater treatment plants-WTP I: G0–GB4, WTP II: O–B4, WTP III: XO–XB4, WTP IV: HO–HB2; red line indicates medium risk).

In this study, the risks assessment to PFASs for aquatic organisms was conducted based on the risk quotient (RQ) method (Figure 3).

RQ parameters greater than 1 indicate that the tested sediment/water could pose a high risk to the aquatic environment, and such values were not observed in the evaluated sampling points. The logarithmic RQ values for investigated pollutants are presented in Figure 3. In the case of sediment samples, RQ values were lower than 0.1 in all tested samples. Based on the calculated RQ values, the environmental risks resulting from all PFASs seemed to be negligible, indicating low or medium (five sampling points of aquatic phase) risk of PFASs to aquatic organisms. Medium risk was estimated for sampling points W4 (PFBS, RQ = 0.17), W10 (PFDoA, RQ = 0.19), W25 (PFDoA, RQ = 0.32), A3 (PFDoA, RQ = 0.13), and HA3 (PFDoA, RQ = 0.27).

## 3. Discussion

The results obtained in this study were compared with PFASs concentrations in sea sediments around the world. The relative data is presented in Table S1.

Data analysis shows that in the case of samples collected around the discharge collectors, the values are comparable to the values in sea sediments reported by other authors. Studies for PFASs content in the estuarine and coastal areas of the East China Sea conducted by Yan et al. (2015) showed that ΣPFAS does not exceed 34.8 ng/g d.w. [18]. Other research conducted in China (the Jiulong Estuary-Xiamen Bay) indicates the presence of PFASs at lower levels (ΣPFAS—3.0–5.4 ng/g d.w.) [19]. Furthermore, in other areas, ΣPFAS was up to a few ng/g d.w. [4,11,17,20,21,22]. In the case of samples taken at the mouth of the Vistula, the recorded values are higher (average Σ17PFAS = 77 ng/g d.w.) but lower than those obtained by Bai et al. (2021) for the area of the Truckee River and Las Vegas Wash (average Σ17PFAS = 272.9 and 345.7 ng/g d.w., respectively) [23]. However, it should be noted that it is impossible to fully compare the results obtained in our research to the results obtained by other authors due to the different amount of tested compounds.

**Figure 4:**
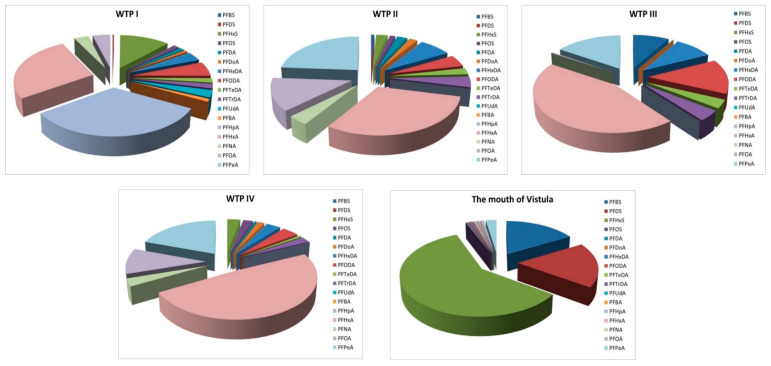
Percentage composition of the PFASs in samples taken around the sewage collectors’ outlets from wastewater treatment plants (WTP I-WTP IV) and from the mouth of Vistula (PFBA-perfluoro-*n*-butanoic acid, PFPeA-perfluoro-*n*-pentanoic acid, PFHxA-perfluoro-*n*-hexanoic acid, PFHpA-perfluoro-*n*-heptanoic acid, PFOA-perfluoro-*n*-octanoic acid, PFNA-perfluoro-*n*-nonanoic acid, PFDA-perfluoro-*n*-decanoic acid, PFUdA-perfluoro-*n*-undecanoic acid, PFDoA-perfluoro-*n*-dodecanoic acid, PFTrDA-perfluoro-*n*-tridecanoic acid, PFTeDA-perfluoro-*n*-tetra-decanoic acid, PFHxDA-perfluoro-*n*-hexadecanoic acid, PFODA-perfluoro-*n*-octadecanoic acid, PFBS-potassium perfluoro-1-butanesulfonate, PFHxS-sodium perfluoro-1-hexanesulfonate, PFOS-sodium perfluoro-1-octanesulfonate, PFDS-sodium perfluoro-1-decanesulfonate).

Percentage composition of the PFASs was calculated as the percent compositions of individual PFASs in the total content of PFASs. Analysis of percentage composition allows the indication that in samples gathered near discharge collectors, the highest percentages was observed for PFHxA (PFHpA > PFHxA > PFHxS > PFOA > PFNA > other for WTP I; PFHxA > PFPeA > PFOA > PFNA > other for WTP II; PFHxA > PFPeA > PFODA > other for WTP III; PFHxA > PFPeA > PFOA > other for WTP IV). However, in the case of samples collected at the mouth of the Vistula, the largest percentage was observed for PFHxS (PFHxS > PFDS > PFBS > other) (Figure 4).

Additionally, identification of the potential source of PFASs in sediment samples was conducted using Pearson correlation analysis (Table 1, Table S2) and other statistical analyses. He at al. (2018) indicate that PFASs could possibly originate from the same sources if a statistically significant correlation among them can be observed [15]. Based on similar characterization of correlation between sampling points/concentration of PFASs, the research area was divided into two zones: Zone I = WTP I, WTP II, WTP III and WTP IV collector and Zone II = the Vistula River.

The composition of the PFASs in the samples taken around the collectors is similar between the various samples, while in the case of the samples taken at the Vistula river mouth, the composition is completely different. In all four wastewater treatment plants, mechanical and biological processes as well as chemical support of phosphorus removal are used, mainly in order to remove nutrients. PFASs are resistant to degradation and removal through the existing biological treatment methods and are thus considered non-degradable [9]. Although long-chain PFASs should be expected in the sediments, since short-chain PFASs are relatively hydrophilic compared to long-chain PFASs [24], the largest share of PFASs in the sediments collected around the collectors comprised PFHxA (C6), PFPeA (C5), PFHpA (C7), and PFOA (C8). PFHxA was present at the highest concentration of all labeled PFASs. Moreover, In Zone I, the strongest correlation was observed for PFPeA and PFHxA, PFDoA and PFNA, and PFHxS and PFOS with PFNA and with PFDoA (Table 1). PFHxA is used as an alternative to PFOA in the production of packaging materials for food/pharmaceutical products and water–oil repellent paper coating, because PFHxA is less toxic than PFOA [21,24]. PFPeA may be used to keep food from sticking to cookware and may also be used in some food packaging. PFHxA and PFPeA can be released to the Gulf of Gdansk with treated wastewater. Research conducted by Kim et al. (2012) on discharge of perfluorinated compounds from wastewater treatment plants in Korea showed that in the case of treated wastewater from domestic sources, the highest concentrations were observed for PFHxA and PFOA. Moreover, in the case of one wastewater treatment plant, the concentration of PFHxA and PFOA in treated wastewater was higher than in untreated wastewater [25]. In contrast, in studies conducted by Gallen et al. (2018) in the effluent samples taken from 14 WTPs in Australia, PFHpA and PFHxS were detected [2]. PFHxA, PFPeA, and PFOA were also determined in treated sewage collected from three sewage treatment plants located in Gauteng Province, South Africa, and the efficiency of removing these compounds depended on the treatment processes [8]. PFHxA, PFOA, and PFOS were also the dominant PFASs in the effluents retrieved from WTPs in Sweden [26]. PFHpA, PFOS, and PFOA were the most frequently detected and predominant PFASs in surface sediment samples collected from estuarine and coastal areas of the East China Sea [18].

Pearson correlation analysis has shown that specific patterns were observed for some of the compounds (Table 1), for example for PFHxA and PFHpA (Zone II: r = 0.93), which might indicate an on-going transition to shorter PFASs [13]. These correlations might suggest that PFASs come from similar sources or transfer mechanisms [15]. Among the investigated pollutants, short-chained PFASs (e.g., PFBA) are used as a substitute for PFOA and PFOS and related compounds or are the degradation products of their precursors [27]. A possible source of this pollutant could also be a pharmaceutical company and some food-packing manufacturing plants in the province. Based on the ratios of PFHpA to PFOA in each of the zones, both sources of pollutants can be observed (direct emissions from WTP and accumulation in sediment; the contribution of atmospheric deposition) [28]. PFNA is mainly derived from the production of perfluorinated carboxylic acid, but there are no significant sources in the investigated area, and it might be the effect of deposition processes [29]. The lowest impact on correlation coefficient was observed for sampling point WTP III due to relatively low levels of pollutants (Table S2).

Additionally, to investigate the sources of PFSAs contamination of the Gulf of Gdańsk, the percentage composition of pollutants was analyzed. As previously mentioned, in the case of samples collected around the WTP II-IV collectors, the highest percentage concentration was observed for PFHxA and PFPeA, while in the case of WTP I, the largest percentage was observed for PFHpA and PFHxA. The potential point sources of PFHpA may be a former landfill and an airport [6]. Wastewater treatment plant I is located in Gdańsk, where one of the largest airports in Poland is situated.

In the case of samples taken from the mouth of the Vistula, the highest ratio was observed for PFHxS, PFDS, and PFBS. The sorption potential of PFASs to sediments (with respect to their structural characteristics and functional group heads) indicates that sorption increases as carbon chain length increases, and that perfluorosulfonates have increased sorption potential compared to their carboxylate counterparts [2]. The Vistula River waters carry pollutants adsorbed on the suspended material from Poland (the basin covers 54% of the area of Poland), Ukraine, Belarus, and Slovakia [30]. All pollutants eventually end up in the Baltic Sea with the Vistula waters. The highest observed percent composition of PFHxS could be associated with the usage of this compound as an alternative to PFOS in many applications including coated textiles, water-repelling paper, and firefighting foams [31]. In research conducted in 2005 by Falandysz et al. (2012), focused on the determination of PFOS, PFHxS, and PFBS in samples collected in the area of the Vistula estuary, the results have indicated that the highest percentage was related to PFHxS and PFOS [16]. River sediment studies for PFASs content by Lam et al. (2016) in Vietnam showed that PFHxS (mean = 2.48 ng/g d.w.) and PFOS (mean value = 0.52 ng/g d.w.) were the two predominant PFASs in sediment samples, accounting for 71% and 15% of the total PFAS concentration, respectively [32]. PFOS is commonly used in electroplating and anti-fog agents in industry [33]. In research conducted by Becanova et al., 2016, on riverbed sediment samples from an industrial region in the Morava River catchment in the Czech Republic, the highest detection frequencies as well as the highest concentrations in riverbed sediment samples were observed for PFASs with six to eight carbon atoms per molecule (PFHxS, PFHpS, and PFOS, respectively) [31].

In the case of samples taken from the mouth of the Vistula, high percentages were also observed for PFBS and PFDS. PFBS, with a short chain perfluoroalkylsulfonate having four carbons, has many general physiochemical properties that are similar to its longer chain-counterparts, such as the six carbon perfluorohexanesulfonic (PFHxS) and eight carbon perfluorohexanesulfonic acid (PFOS) or like other perfluoroalkylsulfonates, which are amongst the most stable and persistent organic molecules [34]. PFBS is used for production of surfactants, pesticides, and flame-retardants [35]. PFBS, PFHxS, and PFOS have also been determined in sediments of the Bering Shelf and the Chukchi Sea. The largest ratio was observed for PFOS (35%) followed by PFHxS and PFBS (16% each) [22]. The results of other sediment studies by Kahkashan et al. (2019) in the Chukchi Sea, the Bering Sea, and Arctic Ocean area indicated that PFBS, PFOA, and PFOS were the dominant PFASs in these areas [24]. Research by Zhao et al. (2015) indicates that PFOS was the dominant compound in marine sediments in the German Bright, and the enrichment of PFOS in sediment might be strongly related to the compound structure itself [11]. In 2013, Filipovic et al. conducted a mass balance study in the Baltic Sea for selected PFASs (PFOA, PFDA, PFOS, and PFHxA) [26]. They found that river inflow and atmospheric deposition were the dominant inputs, while wastewater treatment plant effluents made a minor contribution (<5%). For PFOS, the input to the Baltic Sea was dominated by riverine discharge (77%), with a lesser contribution from atmospheric deposition (20−21%). For PFHxA, PFOA, and PFDA, riverine discharge accounted for 10−73%, 48−59%, and 28−67% of the input, respectively. Another important source was atmospheric deposition (11–37% for PFHxA, 34–43% for PFOA, 31–72% for PFDA, and 20–21% for PFOS). The transport of PFASs in the atmosphere most likely takes place via two routes. Nonionic precursor compounds such as fluorotelomer alcohols (FTOHs) are transported in the gas phase where they can undergo transformation before being deposited as PFAAs and the ionic PFAAs can undergo atmospheric transport sorbed to airborne particles [36].

Moreover, on the basis of the correlation analysis, the strongest relation can be observed for PFOA and PFHpA, PFNA and PFOA, PFTeDA and PFHxDA with PFDoA, and PFHxDA and PFTeDA. As previously mentioned, the Vistula River is a receiver of pollutants both from diffuse and point sources. Among others, treated wastewater from a treatment plant (a sewage treatment plant in the capital of Poland, Warsaw) is released to the waters of the Vistula River. Therefore, it is not possible to clearly define the sources of PFASs.

The spatial distribution of pollutants may also result from the hydrodynamic situation in the mouth of the Vistula area. The estuary of the Vistula River is characterized by a complicated circulation of water [37]. This is due to interaction of the current of the flowing river and the currents occurring in its foreland, in the Gdansk Bay, as well as the mixing of salt and fresh waters typical of an estuary [38]. After exiting the estuary, the waters of the Vistula flow mainly in the east and north-east directions, and to a lesser extent in the north-west and west directions [37], which was reflected in the research results. Higher concentrations of PFASs were observed mainly in the eastern direction (for samples W4, W6, and W8) and the north (for samples W10, W16, and W27). The exception were the samples W12 and W13, which are located west of the mouth of the Vistula. Sample W12 was collected close to the shoreline, so the results may be influenced by direct runoff from land. It should also be noted that the bathymetry of the Vistula estuary is diversified. To the east of the mouth of the Vistula, the shoreline is almost straight, while to the west it is much more varied. An important element in the bathymetry of the Vistula estuary area is the alluvial cone, constituting the new outer delta. It is made up of shallows on both sides of the estuary and an island—a sandbank in the very estuary, made of alluvial material (sand and gravel) transported by the waters of the Vistula. The cone is a serious obstacle to the currents along the shore, becoming a spur that catches the material carried by said currents [37].

**Figure 5:**
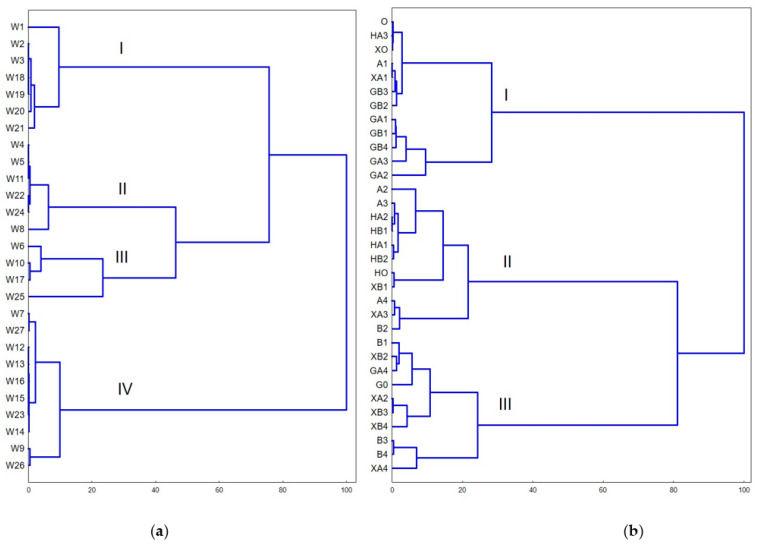
Dendrogram showing cluster analysis results for location sediment samples changes for the mouth of the Vistula (a) and the sewage collectors’ outlets from WTPs (b) (the Ward’s method, 1-rPearson distance) (I–IV-clusters).

To investigate the relationship between different sampling points, cluster analysis was conducted. In the case of samples taken from the mouth of the Vistula, we can distinguish four clusters (Figure 5). The first cluster comprises samples in which PFSAs compounds were not determined (except for sample W1 in which PFBS and PFDS were determined at very low levels—0.066 ± 0.011 and 0.072 ± 0.013 ng/g d.w.). These samples also had the lowest ∑PFAS content. Cluster II contains samples with a high concentration of PFBS. At the same time, in these samples compounds from the PFSAs group (except for PFDS in W8 sample) and PFUdA and PFBA were below the limit of quantification.

In the case of samples collected around the discharge collectors, some samples from nearby locations were clustered together, for example from WTP I, which are located in the I cluster or WTP IV, which are located in cluster II (Figure 5). Moreover, the first cluster contains samples 0, A1, HA3, X0, and XA1, which are characterized by the highest concentration of PFHxA. In samples grouped in cluster II, PFOA, PFTrDA, and PFHxDA were determined, while in samples located in cluster III, PFOD and PFHxDA were predominant. However, some samples from nearby locations are in different clusters, for example, those collected at WTP II, which means that there is a certain variability in PFASs concentrations.

Some authors indicate that the content of organic carbon and grain size have an influence on PFASs concentration distribution in sediments [13,22,31], so this relationship has also been analyzed. Sediment characteristics are presented in Table S3. The samples taken around the collectors are mostly sandy. The exception are some samples in WTP I and WTP III, which are silt. Samples taken at the Vistula estuary included sand of various graining and silt. The relationship between type of sediments and ΣPFAS was analyzed. No dependency between these parameters was observed. At the same time, the study results indicate that the concentration of PFASs increases with increasing organic fraction content. However, the available data is insufficient to clearly state this relationship and it is necessary to conduct further research in this direction.

Research conducted by Pan and You (2010) and You et al. (2010) indicates that PFSAs could be increasingly sorbed on suspended particles and settle to the sediment as salinity increases [12,39]. The influence of the salting-out effect may also influence the results obtained in our research for the area of the Vistula estuary. Samples W1–W3, W5, and W11, which are located closest to the riverbed, have a low content of PFSAs. With increasing distance from the location where the river flows into the Gulf of Gdansk, the content of PFSAs increases. However, it should be mentioned that the circulation of water has an impact on the spatial distribution of pollutants in this area, as was previously described.

Comparing RQ values obtained for the Gulf of Gdansk with other aquatic ecosystems, a generally similar pattern can be observed (mainly RQ < 0.1) [40]. Nevertheless, uncertainty might disturb correct assessment of the environmental risk resulting from PFASs and their real impact on aquatic organisms due to the scarcity of toxicity data for these contaminants and a lack of environmental data on the levels of pollutants (e.g., risk parameters are derived using test organisms or endpoints that might not be sensitive to PFASs, non-standardized toxicity methods, limited toxicological data for marine organisms (especially sediment), in case of secondary poisoning of predators problems with using biomagnification factors as indicators of the bioaccumulation potential due to unexpected accumulation patterns of PFAS, diversity of samples, unavailability of spatial and temporal monitoring data) [41]. Currently, risk assessments conducted within regulatory frameworks are based on monitoring (variable scale in different countries) and developing effects-based thresholds/benchmarks to support retrospective assessments. The established thresholds for the effects of PFASs are available only for selected compounds in the elements of the environment. Parameters are mainly estimated for direct effects of PFOS in freshwater aquatic species [42]. In the European Union (EU), several thresholds of PFOS are available. Additionally, from 2013, the above mentioned compound was included in the amended list of priority substances of Directive 2000/60/EC as set forth in Directive 2013/39/EU with set values of the Environmental Quality Standard (EQS; annual average EQS = 0.65 ng/L; maximum allowable concentration EQS = 36 mg/L) for surface water. In the study area only in the HA3 sample (WTP IV), PFOS concentration (1.2 ng/L) was higher than the annual average EQS [43]. However, there are no published benchmarks for sediments due to limited research on effects in benthic invertebrates after direct exposure to PFAS. The EU established that insufficient data are available to confirm the need for a sediment quality standard or to derive a threshold, thus electing not to set a value. Norwegian sediment thresholds (e.g., no-effect threshold = 220 μg/kg d.w.) were proposed for PFOS in marine sediments, and they may also provide some basis for screening-level risk decisions for freshwater sediments (all estimated values were lower than threshold) [42,44,45,46].

For the collected samples differences were observed in the assessment of the toxicity with two different biotests. For example, sample H0 was toxic to the Ostracoda and non-toxic to *A. fischeri*. Ostracoda, used as indicator organisms in the chronic toxicity test, are small crustaceans, which have a fully developed gastrointestinal tract, through which toxic substances can enter into the organism (easily bioavailable pollutants can also enter via body shells and gills) [47]. These organisms live in the upper sea floor layer, mainly in the zone of aquatic vegetation and bottom sediments, feeding on detritus and algae. Consequently, ostracoda are sensitive to both hydrophilic and hydrophobic pollutants. On the other hand, *Aliivibrio fischeri* bacteria are sensitive to the compounds contained in the aqueous extract. In order to look for the relationship between ecotoxicity and PFASs, cluster analysis was conducted. No linear relationship was observed between the content of PFASs and ecotoxicity in samples collected both around the collectors and at the mouth of the Vistula. This might be also due to the applied test not being sensitive enough. This indicates that the toxicity may be influenced by other compounds in the samples.

## 4. Materials and Methods

### 4.1. Research Area Characteristic

The Gulf of Gdansk is a southeastern bay of the Baltic Sea. This area is particularly exposed to pollution due to a high degree of urbanization and industrialization. The sources of pollution in this area are industry, mainly in the energy sector, and dynamically developing road transport. Moreover, the route of ship traffic to the ports of Gdynia and Gdansk runs through the Bay of Gdansk. The waters of the Gulf of Gdansk are also a place for depositing sediments from dredging fairways and reservoirs. At the same time, four wastewater treatment plants located on the coast are discharging treated wastewater into the waters of the Gulf.

Two of these wastewater treatment plants are units that collect wastewater from the Tri-City agglomeration (Gdansk, Gdynia, and Sopot), and the other two are regional plants. The waters of the Vistula River, which carry pollution from other regions of Poland, also have an impact on the contamination of the Bay of Gdansk waters. The Vistula River length is 1047.5 km, and the catchment covers 194,424 km^2^. The river basin covers 54% of the area of Poland, and almost 60% of Poland’s population lives in the area of the basin (around 22.9 million people) [30].

### 4.2. Sample Collection

**Figure 6:**
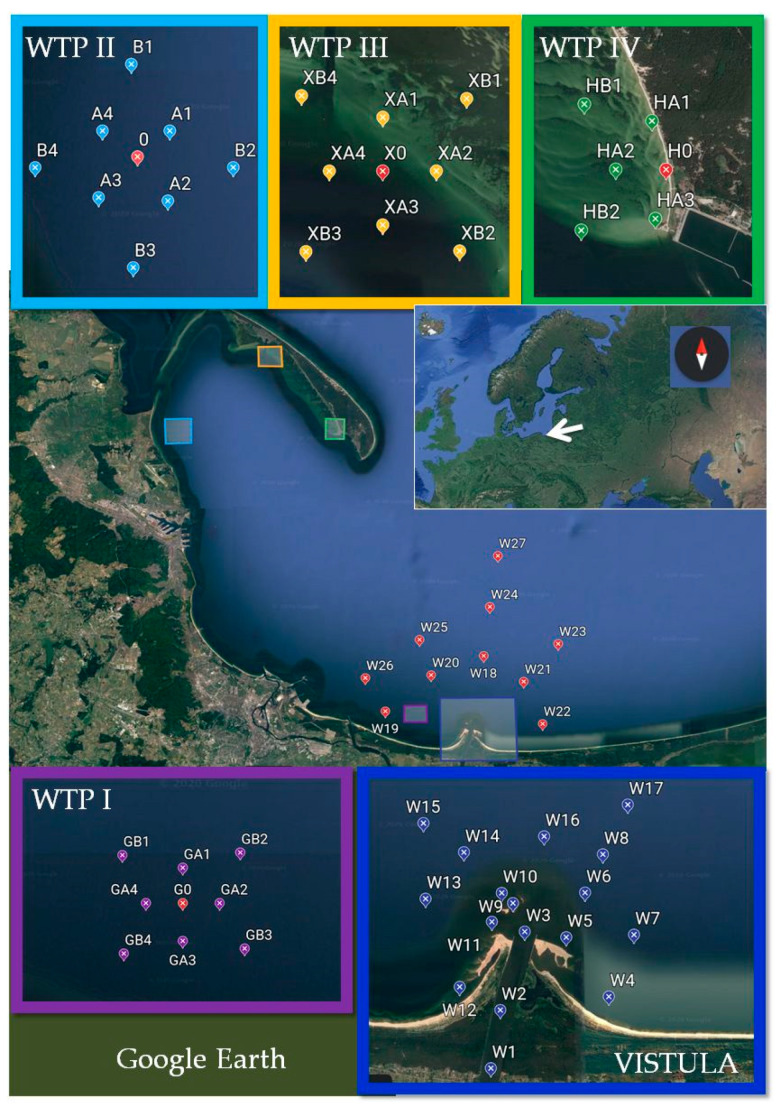
Sampling sites of sediments in the area the Gulf of Gdansk (WTP I–IV-wastewater treatment plant).

In this study, 33 sediment samples were collected around the wastewater collector outlets from WTPs discharging treated wastewater into the Gulf of Gdansk. Two of the WTPs (I and II) are among the largest in the region, while the other two (III and IV) are local, with lower efficiency. Twenty-seven sediment samples (W1–W27) were taken at the estuary of the Vistula River (Figure 6). Surface sediments were taken with Van Veen’s grab, transported to laboratory, and frozen (−20 °C). Subsequently they were lyophilized (Scanvac CoolSafe 110-4 PRO—Lynge, Denmark).

### 4.3. Analytical Procedure

#### 4.3.1. Toxicity Test

Due to the fact that chemical measurements are not able to reliably predict the impact of complex chemical mixtures on ecosystems, it is reasonable to complement such information with the results of ecotoxicological studies [47]. To assess the toxicity of collected sediments, two bioassay tests were applied: Microtox (Modern Water Inc.—New Castle, DE, USA) and Ostracodtoxkit F^TM^ (MicroBioTests Inc.—Gent, Belgium).

The acute toxicity of sediment aqueous extracts has been assessed using the Microtox test, which utilizes the marine bacteria *Aliivibrio fischeri* according to the ISO 11348:2-2002 procedure [48]. This test is an in vitro testing system that uses bioluminescent bacteria to detect the toxic effect of substances present in different parts of the environment. Response to toxicity of chemical compounds is observed as a change in luminescence, which is measured with a Microtox Model 500 analyzer (Strategic Diagnostics Inc.—Newark, DE, USA). This sensitive photometer is integrated with an incubator, which allows the conducting of measurements under constant conditions (15 °C). The reduction in the emission of light radiation is proportional to the concentration of pollutants present in the sample. The determination of toxicity is based on the protocol “81.9% Basic Test”, which is the only available protocol in the *MicrotoxOmni^TM^* software (AZUR Environmental—Carlsbad, CA, USA). The reference test material used in Microtox assay was zinc sulfate, which is used to identify the sensitivity of the bacteria. According to the producer’s recommendations, after a 15-min incubation period, the EC50 of the control solution should be in the range of 3.0–10.0 mg/L. The obtained reference test material results were consistent with the above guidelines.

Due to the fact that the used ecotoxicological test is addressing toxicity of contaminants released to the water phase, the preparation of an aqueous extract consisted in measuring 10 g of the sediment and then adding 40 mL of demineralized water. Sediment samples were mixed and shaken for 18 h at 20 °C. Afterwards, the water extracts were centrifuged for 10 min at 5000 rpm (Eppendorf Centrifuge 5804—Hamburg, Germany) and decanted for toxicity analysis. For the Microtox assay, the pH of the sediment suspensions was measured for each sample and if needed adjusted within the required range of 6.5–8.0 [47,49].

*Ostracodtoxkit F^TM^ is* used to assess the sub-chronic toxicity of sediments using the crustacean *Heterocypris incongruens* (Ostracoda). The toxicity assay is based on two distinct effect criteria: mortality of the test organisms or growth inhibition, resulting from the direct contact with the (non-diluted) solid sample. Growth inhibition is investigated by measuring the length of live Ostracoda after the end of the incubation period (six days) and compared with the mean value calculated on the day the test was started. The obtained results are then compared with the result of growth increase measured in a control sample. Growth inhibition is determined only for sediments for which less than 30% mortality has been found [50]. The test is run according to the standard operational procedure supplied by the producer. Acceptability criteria for the test are mortality <20% and mean growth increment exceeding 400 μm in the reference sample.

In both bioassays, according to Persoone et al. (2003), the following toxicity criteria were adopted: non-toxic samples, percentage effect (PE) < 20%; slightly toxic samples, 20% ≤ PE < 50%; toxic samples 50% ≤ PE < 100%; and highly toxic samples, PE = 100% [51].

#### 4.3.2. Chromatographic Analysis

After lyophilization, sediment samples were prepared according to Gałęzowska et al. 2020 [52]. Approx. 5 g of dry sediment was weighed. All samples have been alkalized and internal standards (ISs) were added. Samples have been extracted with methanol and cleanup on SPE column (HR-XAW column, Macherey-Nagel—Düren, Germany). Solvent extracts have been analyzed using ultrahigh-performance liquid chromatography and tandem mass spectrometry (Nexera X2UHPLC-MS/MS, Shimadzu Corp.—Kyoto, Japan). Electrospray ionization in negative ion mode tandem mass spectrometry was used. Chromatographic separation was performed using an Acquity UPLC BEH C18, 1.7 µm, 2.1 mm, and 100 mm analytical column (Waters—Milford, Massachusetts, MA, USA). The mobile phase consisted of a linear gradient (methanol and ammonium acetate in water) at a flow rate of 0.6 mL/min.

The following 17 PFASs were determined: 13 carboxylic and 4 sulfonic acids (perfluoro-*n*-butanoic acid—PFBA, perfluoro-*n*-pentanoic acid—PFPeA, perfluoro-*n*-hexanoic acid—PFHxA, perfluoro-*n*-heptanoic acid—PFHpA, perfluoro-*n*-octanoic acid—PFOA, perfluoro-*n*-nonanoic acid—PFNA, perfluoro-*n*-decanoic acid—PFDA, perfluoro-*n*-undecanoic acid—PFUdA, perfluoro-*n*-dodecanoic acid—PFDoA, perfluoro-*n*-tridecanoic acid—PFTrDA, perfluoro-*n*-tetra-decanoic acid—PFTeDA, perfluoro-*n*-hexadecanoic acid—PFHxDA, perfluoro-*n*-octadecanoic acid—PFODA, potassium perfluoro-1-butanesulfonate—PFBS, sodium perfluoro-1-hexanesulfonate—PFHxS, sodium perfluoro-1-octanesulfonate—PFOS, sodium perfluoro-1-decanesulfonate—PFDS). The method precision range was 0.29–9.8% for all PFASs. Recoveries of extraction were acceptable, within a range of 60–105% for all PFASs (more details in Gałęzowska et al. 2020 [52]). Additionally, method quantification limit (MQL) was estimated, which is determined on the basis of the limit of quantitation (analyte concentration that yields a measured peak with signal to noise ratio of 10).

#### 4.3.3. Environmental Risk Assessment

Risk quotient (RQs) values for the impact of PFASs in sea sediment and water were calculated for algae and invertebrates by comparing the measured environmental concentration (MEC) in the sea samples to the predicted no-effect concentration (PNEC) according to Equation (1) [53,54]:

(1) $$\mathrm{RQ}\;=\;\frac{{\mathrm{MEC}}_{x}}{{\mathrm{PNEC}}_{x}}$$

where:

MEC—measured environmental concentration [ng/L or ng/kg d.w.—dry weight]

PNEC—predicted no effect concentration [ng/L or ng/kg]

*x*—values for water or sediment.

To estimate PNECs values (Equation (2)), the lowest recorded toxicity data for all species of aquatic animals were collected from the literature [55]. Because of insufficient data on toxicity of PFASs to sediment, the equilibrium partitioning method (EqPM) was applied to calculate the PNEC_sediment_ from PNEC_water_ according to the Technical Guidance Document of the European Union [55,56]. PNEC_sediment_ values used in risk assessment are listed in Supplementary Materials Table S4.

(2) $$\mathrm{PNEC}\;=\;\frac{\mathrm{NOEC}\;\mathrm{or}\;L\left(E\right)C50}{AF}$$

where:

NOEC—no observed effect concentration

EC50—the concentration of compound at which the organism gives half-maximal response

LC50—the concentration of compound where 50% of the organisms die

*AF*—an appropriate standard assessment factor (the assessment factor value is corresponded with availability of number of trophic levels of NOEC).

The partitioning behavior of PFASs between sediment and surface water is crucial to evaluate the transport of perfluorinated compounds in the aquatic environment. The distribution coefficient (K_d_ = C_s_/C_w_; where C_s_ and C_w_ are concentrations of PFASs in sediment [ng/kg d.w.] and surface water [ng/L]) is a commonly used parameter in the evaluation of organic compounds partitioning in the environment. The prediction of PFASs concentration in the aqueous phase (C_w_) was obtained by using the ratio of the measured concentration in the sediment to the distribution coefficient (K_d_). The values of coefficient parameters were estimated by Gebbink et al. 2016 during research conducted in the Baltic sea area and have been used in the presented risk assessment [20]. In case of missing research data, the coefficient parameter K_d_ was estimated by multiplying the fraction of organic carbon (f_oc_ [%]) in the sediment samples and the organic carbon partitioning coefficient (K_oc_) [57,58]. The K_oc_ data were obtained using the EPI (Estimation Programs Interface) Suite™ software by the U.S. Environmental Protection Agency [59] (see Table S4 in Supplementary Materials [60,61,62,63,64,65,66,67]).

The environmental risk characterization of perfluorinated compounds in the aquatic environment was performed using the following ranking criteria [68]:

○ 0.01 ≤ RQ < 0.1  -> “low risk”
○ 0.1 ≤ RQ < 1  -> “medium risk”
○ 1 ≤ RQ   -> “high risk”

## 5. Conclusions

This investigation has shown that:

- Concentrations of Σ17PFSAs varied between 0.00403 ± 0.0073 and 40.6 ± 2.5 ng/g d.w. for samples taken around the discharge collectors and between 0.509 ± 0.017 and 614 ± 46 ng/g d.w. for samples taken at the Vistula estuary.
- Percentage composition of the PFASs depended on the sampling location; in the case of samples collected around the wastewater treatment plant outlets, the percentage composition is similar, while it differs for samples collected at the mouth of the Vistula.
- In the case of samples taken around the discharge collectors, where long-chain PFASs were expected, the largest percentage composition was observed for PFHxA (C6), PFPeA (C5), PFHpA (C7), and PFOA (C8); those compounds can be emitted with treated wastewater.
- PFHxS, PFDS, and PFBS dominated in the case of samples collected at the mouth of the Vistula, which is consistent with the studies of other authors on river sediments.
- No toxic effect was observed on the indicator organisms for most of the samples. In several samples, a significant increase in the luminescence of the *Aliivibrio fischeri* bacteria was observed, which may indicate the presence of the phenomenon of hormesis.
- No relationship was observed between ecotoxicity and the content of compounds from the PFASs group.
- The environmental risks resulting from all PFASs seemed to be negligible, indicating low or medium (six sampling points of Vistula’s aquatic phase in the River) risk from PFASs to aquatic organisms.
- For all sediment samples, RQ values were lower than 0.01.
- However, it is necessary to evaluate the environmental risk using new toxicity data if they become available or if accessibility of long term analytical data on pollutants concentration in the environment will increase.

## Figures and Tables

**Table 1:** Correlation coefficient of different PFASs in sediment from the Gulf of Gdansk (correlation is significant at *p* < 0.05, dark green color—strong correlation) (WTP I-IV-wastewater treatment plant, PFBA-perfluoro-*n*-butanoic acid, PFPeA-perfluoro-*n*-pentanoic acid, PFHxA-perfluoro-*n*-hexanoic acid, PFHpA-perfluoro-*n*-heptanoic acid, PFOA-perfluoro-*n*-octanoic acid, PFNA-perfluoro-*n*-nonanoic acid, PFDA-perfluoro-*n*-decanoic acid, PFUdA-perfluoro-*n*-undecanoic acid, PFDoA-perfluoro-*n*-dodecanoic acid, PFTrDA-perfluoro-*n*-tridecanoic acid, PFTeDA-perfluoro-*n*-tetra-decanoic acid, PFHxDA-perfluoro-*n*-hexadecanoic acid, PFODA-perfluoro-*n*-octadecanoic acid, PFBS-potassium perfluoro-1-butanesulfonate, PFHxS-sodium perfluoro-1-hexanesulfonate, PFOS-sodium perfluoro-1-octanesulfonate, PFDS-sodium perfluoro-1-decanesulfonate).

**WTP I—WTP IV**	**PFBA**	**PFPeA**	**PFHxA**	**PFHpA**	**PFOA**	**PFNA**	**PFDA**	**PFUdA**	**PFDoA**	**PFTrDA**	**PFTeDA**	**PFHxDA**	**PFODA**	**PFBS**	**PFDS**	**PFHxS**	**PFOS**
PFPeA	−0.095																
PFHxA	−0.084	0.887															
PFHpA	0.011	−0.130	−0.094														
PFOA	−0.102	0.319	0.086	−0.027													
PFNA	0.124	0.717	0.595	−0.080	0.465												
PFDA	0.082	0.412	0.083	−0.074	0.551	0.540											
PFUdA	0.311	−0.152	−0.091	0.107	−0.100	0.050	0.028										
PFDoA	−0.037	0.744	0.614	−0.090	0.450	0.952	0.573	−0.066									
PFTrDA	−0.073	0.713	0.658	−0.113	0.285	0.407	0.388	−0.159	0.449								
PFTeDA	−0.065	0.712	0.739	−0.003	0.105	0.198	0.263	−0.101	0.226	0.696							
PFHxDA	−0.100	0.639	0.724	−0.088	0.141	0.323	0.162	−0.189	0.343	0.870	0.774						
PFODA	−0.032	0.149	0.164	−0.189	0.168	0.069	0.062	−0.118	0.082	0.337	0.182	0.474					
PFBS	−0.072	−0.097	−0.108	−0.097	−0.109	−0.101	−0.082	−0.119	−0.086	0.417	−0.117	0.275	0.500				
PFDS	−0.045	−0.066	−0.068	0.873	0.034	−0.066	−0.053	−0.071	−0.059	−0.019	0.068	0.032	−0.117	−0.050			
PFHxS	−0.081	0.381	0.296	0.675	0.297	0.483	0.363	−0.089	0.524	0.286	0.216	0.238	−0.037	−0.090	0.798		
PFOS	0.029	0.724	0.649	−0.074	0.370	0.923	0.538	0.049	0.959	0.473	0.242	0.373	0.114	−0.078	−0.049	0.534	
ΣPFASs	−0.091	0.942	0.949	−0.009	0.295	0.698	0.297	−0.121	0.721	0.778	0.745	0.779	0.279	0.006	0.045	0.462	0.736
**VISTULA**	**PFBA**	**PFPeA**	**PFHxA**	**PFHpA**	**PFOA**	**PFNA**	**PFDA**	**PFUdA**	**PFDoA**	**PFTrDA**	**PFTeDA**	**PFHxDA**	**PFODA**	**PFBS**	**PFDS**	**PFHxS**	**PFOS**
PFPeA	−0.182																
PFHxA	−0.123	−0.047															
PFHpA	−0.069	−0.131	0.933														
PFOA	−0.066	−0.149	0.953	0.991													
PFNA	−0.090	−0.153	0.946	0.984	0.991												
PFDA	−0.142	0.051	−0.106	−0.191	−0.183	−0.135											
PFUdA	−0.083	−0.208	0.065	−0.022	0.000	0.038	0.433										
PFDoA	−0.069	−0.180	0.870	0.835	0.854	0.855	0.140	0.082									
PFTrDA	−0.084	−0.226	0.627	0.518	0.543	0.585	−0.032	0.389	0.548								
PFTeDA	−0.102	−0.115	0.808	0.733	0.755	0.762	0.188	0.137	0.967	0.533							
PFHxDA	−0.105	−0.041	0.845	0.761	0.784	0.789	0.198	0.196	0.947	0.490	0.974						
PFODA	−0.078	0.252	0.119	−0.094	−0.065	−0.046	0.590	0.374	0.304	0.032	0.479	0.533					
PFBS	0.731	−0.272	−0.123	−0.079	−0.077	−0.091	0.048	0.354	−0.086	−0.014	−0.071	−0.066	0.065				
PFDS	−0.087	−0.105	0.037	−0.045	−0.039	−0.019	0.549	0.512	0.383	0.057	0.512	0.499	0.741	0.129			
PFHxS	−0.108	0.223	0.100	−0.050	−0.030	−0.029	0.388	0.619	−0.029	−0.067	0.036	0.189	0.576	0.189	0.301		
PFOS	−0.058	0.205	−0.102	0.048	−0.004	0.080	0.231	−0.065	−0.026	−0.062	0.021	0.022	0.049	−0.118	0.019	−0.081	
ΣPFASs	0.029	0.109	0.115	−0.023	−0.004	0.000	0.476	0.718	0.110	−0.013	0.200	0.324	0.699	0.381	0.554	0.939	−0.079

If one detected used as zero in calculating ½ method limit detection (MDL).

## Data Availability

Data contained within the article and supplementary materials are available on request from the authors.

## Supplementary Materials

The following are available online, Figures S1 and S2 and Tables S1–S4.

## Sample Availability

Not available.
