# Supplementary material for: Environmental Risk Assessment Resulting from Sediment Contamination with Perfluoroalkyl Substances
Source: Molecules. 2020 Dec 29;26(1):116. doi: 10.3390/molecules26010116 (PMC7795547; doi:10.3390/molecules26010116)
Supplement: Supplementary file 1 [file molecules-26-00116-s001.pdf]

# Environmental Risk Assessment Resulting from Sediment Contamination with Perfluoroalkyl Substances

**Grażyna Gałęzowska, Justyna Rogowska *, Ewa Olkowska, Wojciech Ratajczyk and Lidia Wolska**

Department of Environmental Toxicology, Faculty of Health Sciences, Medical University of Gdansk, Debowa Str. 23A, 80-204 Gdansk, Poland; grazyna.galezowska@gumed.edu.pl (G.G.); ewa.olkowska@gumed.edu.pl (E.O.); sinclair@gumed.edu.pl (W.R.); lidia.wolska@gumed.edu.pl (L.W.)

* Correspondance: justyna.rogowska@gumed.edu.pl; Tel.: +48-58-349-19-35

**Table S1.** PFASs concentrations in sea sediments around the world.

| Location | Sample | Year | Compounds | Range [ng/g d.w.] | References |
|---|---|---|---|---|---|
| Semi-enclosed bays in Korea | Sediment cores | 2018 | Σ16 PFASs | 0.0067–0.821 | [4] |
| Coastal areas of the North and Baltic Seas | Surface sediments | 2012 | Σ29 PFASs | 0.018–2.6 | [17] |
| German Bight | Surface sediments | 2015 | ∑16PFAS | 0.056–7.4 | [11] |
| the East China Sea | Surface sediments | 2015 | ∑14PFAS | n.d.–34.8 | [18] |
| the Baltic Proper | Surface sediments | 2016 | ∑24PFAS | 1.33[1] | [20] |
| the Jiulong Estuary-Xiamen Bay | Surface sediments | 2019 | ∑24PFAS | 3.0-5.4 | [19] |
| the Seto Inland Sea, Japan | Surface sediments | 2020 | Σ13 PFASs | 0.05–0.67 | [21] |
| the Bering Sea and the western Arctic | Surface sediments | 2020 | ∑9PFAS | 0.06–1.73 | [22] |
| the Truckee River, USA | Surface sediments | 2021 | ∑17PFAS | 272.9[1] | [23] |
| the Las Vegas wash, USA | Surface sediments | | ∑17PFAS | 345.7[1] | |
| the Gulf of Gdańsk | Surface sediments | 2020 | ∑17PFAS | 0.0403–40.6 | This study |
| the Gulf of Gdańsk, the Vistula estuary | Surface sediments | 2020 | ∑17PFAS | 0.509–614 | This study |

[1]average value
d.w.- dry weight.

**Table S2.** Correlation coefficient of different PFASs in sediment from the Gulf of Gdansk (correlation is significant at $p<0.05$, dark green color – strong correlation, individual analysis of sampling points)

| Vistula | PFBA | PFPeA | PFHxA | PFHpA | PFOA | PFNA | PFDA | PFUdA | PFDoA | PFTrDA | PFTeDA | PFHxDA | PFODA | PFBS | PFDS | PFHxS | PFOS |
|---|---|---|---|---|---|---|---|---|---|---|---|---|---|---|---|---|---|
| PFPeA | -0.182 | | | | | | | | | | | | | | | | |
| PFHxA | -0.123 | -0.047 | | | | | | | | | | | | | | | |
| PFHpA | -0.069 | -0.131 | 0.933 | | | | | | | | | | | | | | |
| PFOA | -0.066 | -0.149 | 0.953 | 0.991 | | | | | | | | | | | | | |
| PFNA | -0.090 | -0.153 | 0.946 | 0.984 | 0.991 | | | | | | | | | | | | |
| PFDA | -0.142 | 0.051 | -0.106 | -0.191 | -0.183 | -0.135 | | | | | | | | | | | |
| PFUdA | -0.083 | -0.208 | 0.065 | -0.022 | 0.000 | 0.038 | 0.433 | | | | | | | | | | |
| PFDoA | -0.069 | -0.180 | 0.870 | 0.835 | 0.854 | 0.855 | 0.140 | 0.082 | | | | | | | | | |
| PFTrDA | -0.084 | -0.226 | 0.627 | 0.518 | 0.543 | 0.585 | -0.032 | 0.389 | 0.548 | | | | | | | | |
| PFTeDA | -0.102 | -0.115 | 0.808 | 0.733 | 0.755 | 0.762 | 0.188 | 0.137 | 0.967 | 0.533 | | | | | | | |
| PFHxDA | -0.105 | -0.041 | 0.845 | 0.761 | 0.784 | 0.789 | 0.198 | 0.196 | 0.947 | 0.490 | 0.974 | | | | | | |
| PFODA | -0.078 | 0.252 | 0.119 | -0.094 | -0.065 | -0.046 | 0.590 | 0.374 | 0.304 | 0.032 | 0.479 | 0.533 | | | | | |
| PFBS | 0.731 | -0.272 | -0.123 | -0.079 | -0.077 | -0.091 | 0.048 | 0.354 | -0.086 | -0.014 | -0.071 | -0.066 | 0.065 | | | | |
| PFDS | -0.087 | -0.105 | 0.037 | -0.045 | -0.039 | -0.019 | 0.549 | 0.512 | 0.383 | 0.057 | 0.512 | 0.499 | 0.741 | 0.129 | | | |
| PFHxS | -0.108 | 0.223 | 0.100 | -0.050 | -0.030 | -0.029 | 0.388 | 0.619 | -0.029 | -0.067 | 0.036 | 0.189 | 0.576 | 0.189 | 0.301 | | |
| PFOS | -0.058 | 0.205 | -0.102 | 0.048 | -0.004 | 0.080 | 0.231 | -0.065 | -0.026 | -0.062 | 0.021 | 0.022 | 0.049 | -0.118 | 0.019 | -0.081 | |
| ∑PFASs | 0.028507 | 0.109304 | 0.114818 | -0.02273 | -0.00421 | -0.00042 | 0.476396 | 0.717736 | 0.110026 | -0.01275 | 0.199738 | 0.323694 | 0.698521 | 0.38102 | 0.553845 | 0.939075 | -0.0791 |
| WTP I | PFBA | PFPeA | PFHxA | PFHpA | PFOA | PFNA | PFDA | PFUdA | PFDoA | PFTrDA | PFTeDA | PFHxDA | PFODA | PFBS | PFDS | PFHxS | PFOS |
| PFPeA | 0.188 | | | | | | | | | | | | | | | | |
| PFHxA | -0.312 | -0.144 | | | | | | | | | | | | | | | |
| PFHpA | -0.296 | -0.220 | -0.109 | | | | | | | | | | | | | | |
| PFOA | -0.312 | 0.068 | -0.284 | 0.646 | | | | | | | | | | | | | |
| PFNA | 0.775 | 0.297 | 0.041 | -0.371 | -0.069 | | | | | | | | | | | | |
| PFDA | 0.753 | 0.267 | -0.368 | -0.364 | -0.159 | 0.717 | | | | | | | | | | | |
| PFUdA | 0.052 | 0.370 | 0.201 | -0.439 | 0.080 | 0.492 | 0.552 | | | | | | | | | | |
| PFDoA | 0.095 | 0.288 | -0.243 | -0.327 | 0.195 | 0.207 | 0.122 | 0.094 | | | | | | | | | |
| PFTrDA | 0.361 | 0.346 | -0.501 | 0.359 | 0.540 | 0.283 | 0.295 | 0.106 | -0.191 | | | | | | | | |
| PFTeDA | -0.028 | 0.241 | -0.485 | 0.759 | 0.674 | -0.097 | 0.150 | -0.023 | -0.249 | 0.691 | | | | | | | |
| PFHxDA | 0.130 | 0.251 | -0.577 | 0.590 | 0.730 | 0.156 | 0.205 | 0.018 | -0.150 | 0.819 | 0.881 | | | | | | |
| PFODA | 0.588 | 0.349 | -0.341 | -0.438 | -0.322 | 0.521 | 0.913 | 0.491 | 0.193 | 0.008 | 0.076 | 0.036 | | | | | |
| PFBS | 0.188 | ND | -0.144 | -0.220 | 0.068 | 0.297 | 0.267 | 0.370 | 0.288 | 0.346 | 0.241 | 0.251 | 0.349 | | | | |
| PFDS | -0.188 | 0.125 | -0.345 | 0.899 | 0.691 | -0.297 | -0.267 | -0.370 | -0.288 | 0.629 | 0.885 | 0.799 | -0.349 | 0.125 | | | |
| PFHxS | -0.225 | 0.135 | -0.304 | 0.884 | 0.668 | -0.331 | -0.319 | -0.373 | -0.330 | 0.631 | 0.858 | 0.773 | -0.402 | 0.135 | 0.996 | | |
| PFOS | 0.393 | 0.226 | -0.306 | -0.332 | 0.234 | 0.662 | 0.813 | 0.792 | 0.240 | 0.335 | 0.173 | 0.348 | 0.672 | 0.226 | -0.226 | -0.271 | |
| ∑PFASs | -0.2399 | -0.00084 | 0.051993 | 0.929932 | 0.695549 | -0.14006 | -0.2588 | -0.16639 | -0.36201 | 0.479398 | 0.787606 | 0.658811 | -0.38017 | -0.00084 | 0.880544 | 0.873676 | -0.16593 |
| WTP II | PFBA | PFPeA | PFHxA | PFHpA | PFOA | PFNA | PFDA | PFUdA | PFDoA | PFTrDA | PFTeDA | PFHxDA | PFODA | PFBS | PFDS | PFHxS | PFOS |
| PFPeA | 0.229015 | | | | | | | | | | | | | | | | |
| PFHxA | 0.241 | -0.021 | | | | | | | | | | | | | | | |
| PFHpA | ND | 0.229 | 0.241 | | | | | | | | | | | | | | |
| PFOA | 0.182 | 0.976 | -0.070 | 0.182 | | | | | | | | | | | | | |
| PFNA | 0.189 | 0.938 | 0.007 | 0.189 | 0.975 | | | | | | | | | | | | |
| PFDA | 0.129 | 0.850 | -0.248 | 0.129 | 0.837 | 0.694 | | | | | | | | | | | |
| PFUdA | ND | 0.229 | 0.241 | ND | 0.182 | 0.189 | 0.129 | | | | | | | | | | |
| PFDoA | 0.214 | 0.953 | -0.197 | 0.214 | 0.944 | 0.883 | 0.879 | 0.214 | | | | | | | | | |
| PFTrDA | 0.092 | 0.688 | 0.182 | 0.092 | 0.650 | 0.501 | 0.865 | 0.092 | 0.659 | | | | | | | | |
| PFTeDA | 0.216 | 0.704 | 0.240 | 0.216 | 0.659 | 0.518 | 0.852 | 0.216 | 0.674 | 0.990 | | | | | | | |
| PFHxDA | -0.018 | 0.445 | 0.686 | -0.018 | 0.387 | 0.327 | 0.436 | -0.018 | 0.309 | 0.800 | 0.799 | | | | | | |
| PFODA | 0.326 | 0.723 | 0.434 | 0.326 | 0.657 | 0.558 | 0.747 | 0.326 | 0.664 | 0.926 | 0.962 | 0.850 | | | | | |
| PFBS | -0.014 | -0.121 | -0.293 | -0.014 | -0.181 | -0.156 | -0.206 | -0.014 | 0.091 | -0.367 | -0.344 | -0.425 | -0.277 | | | | |
| PFDS | ND | 0.229 | 0.241 | ND | 0.182 | 0.189 | 0.129 | ND | 0.214 | 0.092 | 0.216 | -0.018 | 0.326 | -0.014 | | | |
| PFHxS | 0.125 | 0.853 | -0.241 | 0.125 | 0.840 | 0.698 | 1.000 | 0.125 | 0.882 | 0.869 | 0.855 | 0.446 | 0.752 | -0.200 | 0.125 | | |
| PFOS | 0.125 | 0.853 | -0.241 | 0.125 | 0.840 | 0.698 | 1.000 | 0.125 | 0.882 | 0.869 | 0.855 | 0.446 | 0.752 | -0.200 | 0.125 | 1.000 | |
| ∑PFASs | 0.277036 | 0.884195 | 0.410694 | 0.277036 | 0.85116 | 0.818956 | 0.734687 | 0.277036 | 0.782383 | 0.813517 | 0.844817 | 0.777442 | 0.91461 | -0.29749 | 0.277036 | 0.740962 | 0.740962 |
| WTP III | PFBA | PFPeA | PFHxA | PFHpA | PFOA | PFNA | PFDA | PFUdA | PFDoA | PFTrDA | PFTeDA | PFHxDA | PFODA | PFBS | PFDS | PFHxS | |
| PFPeA | 0.145 | | | | | | | | | | | | | | | | |
| PFHxA | 0.166 | 0.922 | | | | | | | | | | | | | | | |
| PFHpA | ND | 0.145 | 0.166 | | | | | | | | | | | | | | |
| PFOA | 0.125 | -0.145 | -0.166 | 0.125 | | | | | | | | | | | | | |
| PFNA | ND | 0.145 | 0.166 | ND | 0.125 | | | | | | | | | | | | |
| PFDA | 0.187 | -0.107 | -0.248 | 0.187 | -0.187 | 0.187 | | | | | | | | | | | |
| PFUdA | ND | 0.145 | 0.166 | ND | 0.125 | ND | 0.187 | | | | | | | | | | |
| PFDoA | ND | 0.145 | 0.166 | ND | 0.125 | ND | 0.187 | ND | | | | | | | | | |
| PFTrDA | -0.645 | 0.630 | 0.641 | -0.645 | -0.232 | -0.645 | -0.290 | -0.645 | -0.645 | | | | | | | | |
| PFTeDA | 0.176 | 0.867 | 0.991 | 0.176 | -0.176 | 0.176 | -0.264 | 0.176 | 0.176 | 0.618 | | | | | | | |
| PFHxDA | -0.394 | 0.571 | 0.730 | -0.394 | -0.315 | -0.394 | -0.444 | -0.394 | -0.394 | 0.843 | 0.753 | | | | | | |
| PFODA | -0.185 | -0.082 | -0.046 | -0.185 | -0.325 | -0.185 | -0.546 | -0.185 | -0.185 | 0.082 | -0.052 | 0.259 | | | | | |
| PFBS | -0.885 | -0.223 | -0.266 | -0.885 | -0.177 | -0.885 | -0.260 | -0.885 | -0.885 | 0.478 | -0.283 | 0.184 | 0.411 | | | | |
| PFDS | ND | 0.145 | 0.166 | ND | 0.125 | 0.143 | 0.187 | ND | ND | -0.645 | 0.176 | -0.394 | -0.185 | -0.885 | | | |
| PFHxS | ND | 0.145 | 0.166 | ND | 0.125 | 0.143 | 0.187 | ND | ND | -0.645 | 0.176 | -0.394 | -0.185 | -0.885 | ND | | |
| PFOS | ND | 0.145 | 0.166 | ND | 0.125 | 0.143 | 0.187 | ND | ND | -0.645 | 0.176 | -0.394 | -0.185 | -0.885 | ND | ND | |
| ∑PFASs | -0.00816 | 0.928951 | 0.974507 | -0.00816 | -0.22398 | -0.00816 | -0.3187 | -0.00816 | -0.00816 | 0.760305 | 0.953247 | 0.797972 | 0.096264 | -0.07183 | -0.00816 | -0.00816 | -0.00816 |
| WTP IV | PFBA | PFPeA | PFHxA | PFHpA | PFOA | PFNA | PFDA | PFUdA | PFDoA | PFTrDA | PFTeDA | PFHxDA | PFODA | PFBS | PFDS | PFHxS | |
| PFPeA | 0.195 | | | | | | | | | | | | | | | | |
| PFHxA | 0.200 | 1.000 | | | | | | | | | | | | | | | |
| PFHpA | 1.000 | 0.195 | 0.200 | | | | | | | | | | | | | | |
| PFOA | 0.253 | 0.135 | 0.142 | 0.253 | | | | | | | | | | | | | |
| PFNA | 0.200 | 1.000 | 1.000 | 0.200 | 0.142 | | | | | | | | | | | | |
| PFDA | 0.127 | 0.997 | 0.996 | 0.127 | 0.102 | 0.996 | | | | | | | | | | | |
| PFUdA | 1.000 | 0.195 | 0.200 | ND | 0.253 | 0.200 | 0.127 | | | | | | | | | | |
| PFDoA | 0.200 | 1.000 | 1.000 | 0.200 | 0.142 | 1.000 | 0.996 | 0.200 | | | | | | | | | |
| PFTrDA | 0.259 | 0.949 | 0.951 | 0.259 | 0.441 | 0.951 | 0.935 | 0.259 | 0.951 | | | | | | | | |
| PFTeDA | 0.200 | 1.000 | 1.000 | 0.200 | 0.142 | 1.000 | 0.996 | 0.200 | 1.000 | 0.951 | | | | | | | |
| PFHxDA | 0.249 | 0.968 | 0.970 | 0.249 | 0.379 | 0.970 | 0.957 | 0.249 | 0.970 | 0.998 | 0.970 | | | | | | |
| PFODA | 0.284 | 0.423 | 0.429 | 0.284 | 0.955 | 0.429 | 0.393 | 0.284 | 0.429 | 0.688 | 0.429 | 0.637 | | | | | |
| PFBS | 1.000 | 0.195 | 0.200 | ND | 0.253 | 0.200 | 0.127 | ND | 0.200 | 0.259 | 0.200 | 0.249 | 0.284 | | | | |
| PFDS | 1.000 | 0.195 | 0.200 | ND | 0.253 | 0.200 | 0.127 | ND | 0.200 | 0.259 | 0.200 | 0.249 | 0.284 | ND | | | |
| PFHxS | 0.200 | 1.000 | 1.000 | 0.200 | 0.142 | 1.000 | 0.996 | 0.200 | 1.000 | 0.951 | 1.000 | 0.970 | 0.429 | 0.200 | 0.200 | | |
| PFOS | 0.200 | 1.000 | 1.000 | 0.200 | 0.142 | 1.000 | 0.996 | 0.200 | 1.000 | 0.951 | 1.000 | 0.970 | 0.429 | 0.200 | 0.200 | 1.000 | |
| ∑PFASs | 0.224171 | 0.992073 | 0.992929 | 0.224171 | 0.258006 | 0.992929 | 0.984902 | 0.224171 | 0.992929 | 0.980927 | 0.992929 | 0.991879 | 0.533576 | 0.224171 | 0.224171 | 0.992929 | 0.992929 |

ND = not detected both analytes, if one detected used as zero in calculating ½ method limit detection (MDL)

WTP-wastewater treatment plant

PFBA-perfluoro-*n*-butanoic acid, PFPeA-perfluoro-*n*-pentanoic acid, PFHxA-perfluoro-*n*-hexanoic acid, PFHpA-perfluoro-*n*-heptanoic acid, PFOA-perfluoro-*n*-octanoic acid, PFNA-perfluoro-*n*-nonanoic acid, PFDA-perfluoro-*n*-decanoic acid, PFUdA-perfluoro-*n*-undecanoic acid, PFDoA-perfluoro-*n*-dodecanoic acid, PFTrDA-perfluoro-*n*-tridecanoic acid, PFTeDA-perfluoro-*n*-tetra-decanoic acid, PFHxDA-perfluoro-*n*-hexadecanoic acid, PFODA-perfluoro-*n*-octadecanoic acid, PFBS-potassium perfluoro-1-butanesulfonate, PFHxS-sodium perfluoro-1-hexanesulfonate, PFOS-sodium perfluoro-1-octanesulfonate, PFDS-sodium perfluoro-1-decanesulfonate

**Table S3.** Sediment characteristic

| Sampling area | $f_{oc}$ [%]: min. – max. (average mean)[1] | Type of sediment[2] |
|---|---|---|
| **The Vistula river** | 4.2 – 9.0 (7.7) | medium grain sand and fine grain sand<br>except: silt - W4,W6-W8,W24,W27 and<br>very fine grain sand - W18-19,W25 |
| **WTP I** | 2.1 – 6.1 (2.8) | coarse grain sand – GA2, GB1, GB3<br>medium grain and fine grain sand - G0, GA3, GB2<br>silt - GA1, GB1, GB4 |
| **WTP II** | 2.2 – 5.7 (2.7) | medium grain and fine grain sand |
| **WTP III** | 1.1 – 6.7 (5.1) | medium grain sand and fine grain sand<br>except: silt - XA3, XB2, XB3 |
| **WTP IV** | 1.6 – 6.8 (5.3) | medium grain sand |

$f_{oc}$ - fraction of organic matter
[1]data obtained from previous research in area of the Baltic Sea
[2] sediment samples were classified based on the Unified Soil Classification System

**Estimation of environmental risk parameters**

To estimate the Predicted No Effect Concentration (PNECs) values (Eq. (S1)), the lowest recorded toxicity data for all species of aquatic animals were collected from the literature [54].

$$PNEC = \frac{\text{NOEC or L(E)C50}}{AF} \quad \text{(S1)}$$

where:
NOEC—no observed effect concentration,
EC50—the concentration of compound at which the organism gives half-maximal response,
LC50—the concentration of compound where 50% of the organisms die,
AF—an appropriate standard assessment factor (the assessment factor value is corresponded with availability of number of trophic levels of NOEC).

Because of insufficient data on toxicity of PFASs to sediments, the lowest toxicological values for water phase ($PNEC_{water}$) have been collected from literature (See Table S4). Additionally, in Table S4, assessment factors are presented, which in all cases were estimated as 1000 (the overall AF generally consists of the following 10-fold factors: extrapolation toxic parameter for acute to chronic effects (10×), across species extrapolation (10x), uncertainty in the overall estimation of effects (10×) [61]). For PFTrDA, PFTeDA, PFHxDA and PFODA, experimental values of $PNEC_{water}$ are not available and in these cases Tox-Tram QSAR or advisory guideline values were applied (recommended by the Department of Health and Human Services in North Carolina). To extrapolate $PNEC_{water}$ values for marine samples, an additional 10-fold factor was used. Investigation is still needed regarding ecotoxicological data on aquatic organisms (especially marine water and sediment) to introduce unified threshold values that allow the comparison of different ecosystems. For example, the following values of $PNEC_{water}$ for PFOA used in risk assessment protocols were found in the literature: 1428 ng/L (freshwater), 20,000 ng/L (freshwater), 100,000 ng/L (freshwater), 570,000 ng/L, 119000000 ng/L [54,62–65].

After collection of $PNEC_{water}$ data, the equilibrium partitioning method (EqPM) was used to calculate the $PNEC_{sediment}$, according to the Technical Guidance Document of the European Union (Eq. (S2)) [54,65]. Additionally, for substances with a log $K_{ow}$ >5 the $PNEC_{sediment}$ calculated value needs to be decreased by a factor of 10 [55].

$$PNEC_{sediment} = (0.783 + 0.0217 \times K_{oc}) \times PNEC_{water} \qquad \text{(S2)}$$

where:
$PNEC_{sediment}$—predicted no effect concentration for marine water
$K_{oc}$—the organic carbon partitioning coefficient
$PNEC_{water}$ —predicted no effect concentration for sea water.

Risk quotient (RQs) values for the PFASs occurring in sea sediment and water phase were calculated for aquatic organisms by comparing the measured environmental concentration (MEC) in the sea samples to the predicted no-effect concentration (PNEC) according to equation (S3) [53,54,66]:

$$RQ = \frac{MEC_x}{PNEC_x} \qquad \text{(S3)}$$

where:
MECx – measured environmental concentration [ng/L or ng/kg d.w. – dry weight],
PNECx – predicted no effect concentration [ng/L or ng/kg],
x – values for water or sediment.

**Table S4.** Information on perfluoroalkyl substances used in the environmental risk assessment

| CAS | Compound name | Compound acronym | log Kow [L/kg][1] | log Koc [L/kg][1] | log Kd [L/kg][2] | Type of toxic parameter used to estimate $PNEC_{water}$[3] | AF used to estimate $PNEC_{water}$[3] | $PNEC_{water}$ [ng/L][3] | $PNEC_{sediment}$ [ng/kg][4] |
|---|---|---|---|---|---|---|---|---|---|
| **375-22-4** | perfluoro-n-butanoic acid | PFBA | 2.14 | 1.813 | 0.26[1] | LC50 | 1000 | 110000 | 241316 |
| **2706-90-3** | perfluoro-n-pentanoic acid | PFPeA | 2.81 | 2.464 | 0.91[1] | LC50/EC50 | 1000 | 32000 | 227176 |
| **307-24-4** | perfluoro-n-hexanoic acid | PFHxA | 3.48 | 3.116 | 2.78 | LC50/EC50 | 1000 | 97000 | 2825310 |
| **375-85-9** | perfluoro-n-heptanoic acid | PFHpA | 4.15 | 3.767 | 2.63 | LC50 | 1000 | 7852 | 1002563 |
| **335-67-1** | perfluoro-n-octanoic acid | PFOA | 4.81 | 4.419 | 2.49 | LC50 | 1000 | 1428 | 814300 |
| **375-95-1** | perfluoro-n-nonanoic acid | PFNA | 5.48 | 5.071 | 3.25 | LC50/EC50 | 1000 | 1000000 | 255618796 |
| **335-76-2** | perfluoro-n-decanoic acid | PFDA | 6.15 | 5.722 | 3.95 | LC50 | 1000 | 45 | 51488 |
| **2058-94-8** | perfluoro-n-undecanoic acid | PFUdA | 6.82 | 6.374 | 4.72 | LC50 | 1000 | 8 | 41073 |
| **307-55-1** | perfluoro-n-dodecanoic acid | PFDoA | 7.49 | 7.026 | 3.31 | LC50 | 1000 | 1 | 32024 |
| **72629-94-8** | perfluoro-n-tridecanoic acid | PFTrDA | 8.16 | 7.677 | 3.59 | lack of PNEC – use of ToxTram QSAR value | | 103 | 10644855 |
| **376-06-7** | perluoro-n-tetra-decanoic acid | PFTeDA | 8.83 | 8.329 | 3.19 | lack of PNEC – use of ToxTram QSAR value | | 83 | 38325704 |
| **67905-19-5** | perfluoro-n-hexadecanoic acid | PFHxDA | 10.17 | 9.632 | 8.08[1] | lack of PNEC – use of ToxTram QSAR value | | 77 | 716061729 |
| **16517-11-6** | perfluoro-n-octadecanoic acid | PFODA | 11.51 | 10.94 | 9.38[1] | lack of PNEC – use of recommended value (the Department of Health and Human Services in North Carolina) | | 140 | 26156990202 |
| **375-73-5** | potassium perfluoro-1-butanesulfonate | PFBS | 1.82 | 2.249 | 0.26 | EC50 | 1000 | 372000 | 1723473 |
| **3871-99-6** | sodium perfluoro-1-decanesulfonate | PFDS | 1.01 | 3.552 | 2.00[1] | LC50 | 1000 | 15400 | 1203247 |
| **2795-39-3** | sodium perfluoro-1-hexanesulfonate | PFHxS | 2.35 | 4.855 | 1.34 | EC50 | 1000 | 250000 | 388703550 |

| | | | | | | | | | |
|---|---|---|---|---|---|---|---|---|---|
| **1763-23-1** | sodium perfluoro-1-octanesulfonate | PFOS | 4.49 | 4.855 | 2.94 | LC50 | 1000 | 610 | 948437 |

$K_{ow}$ – octanol water partitioning coefficient; $K_{oc}$ – organic carbon partitioning coefficient; $K_d$ - sediment/water distribution coefficient, NOEC – no observed effect concentration; EC50 – the concentration of compound at which the organism gives half-maximal response; LC50 – the concentration of compound where 50% of the organisms die; AF – assessment factor; PNEC – predicted no effect concentration

[1] data obtained from the EPI Suite™ model, in case of log Kd additionally data on fraction of organic carbon in sediment were used

[2] [20]

[3] [40,41,54,61,62,67,68]

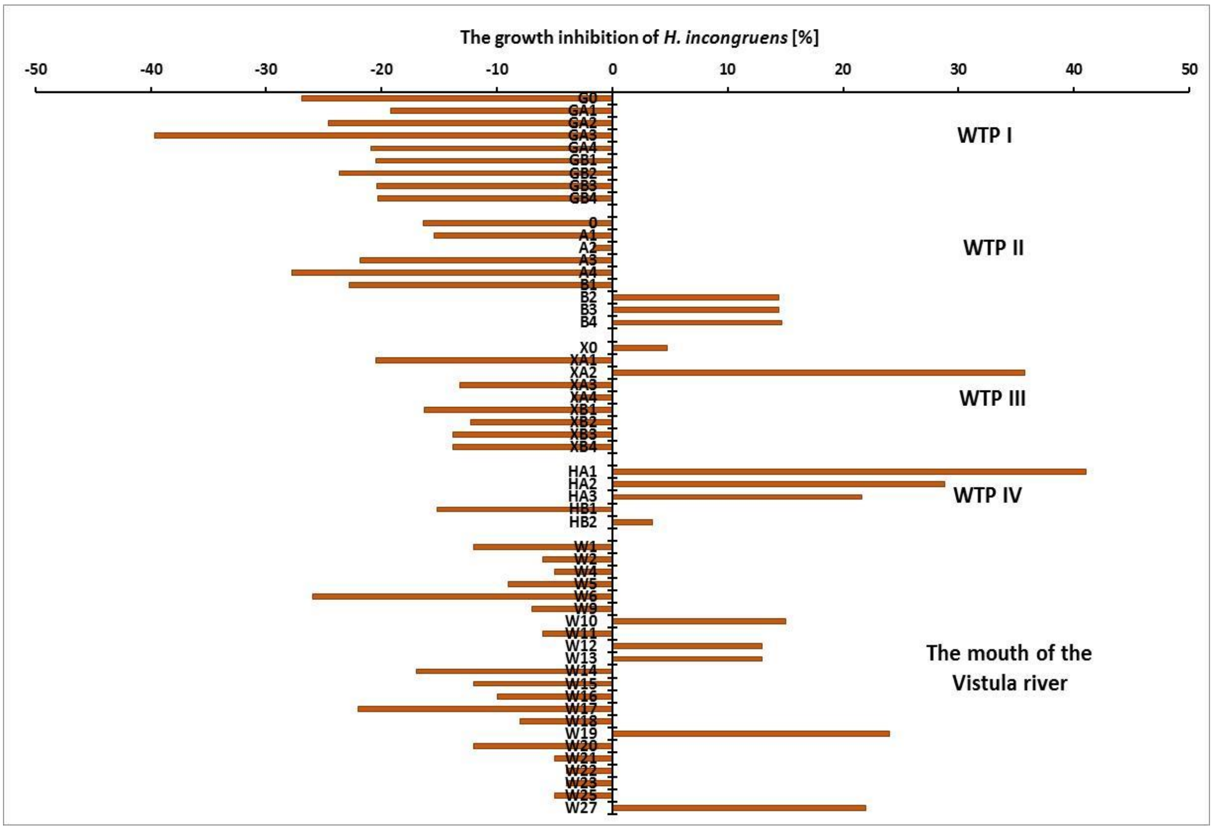


**Figure S1.** The results of the growth inhibition of H. incongruens in samples taken around the sewage collectors' outlets from wastewater treatment plants (WTP I-WTP IV) and from the mouth of Vistula

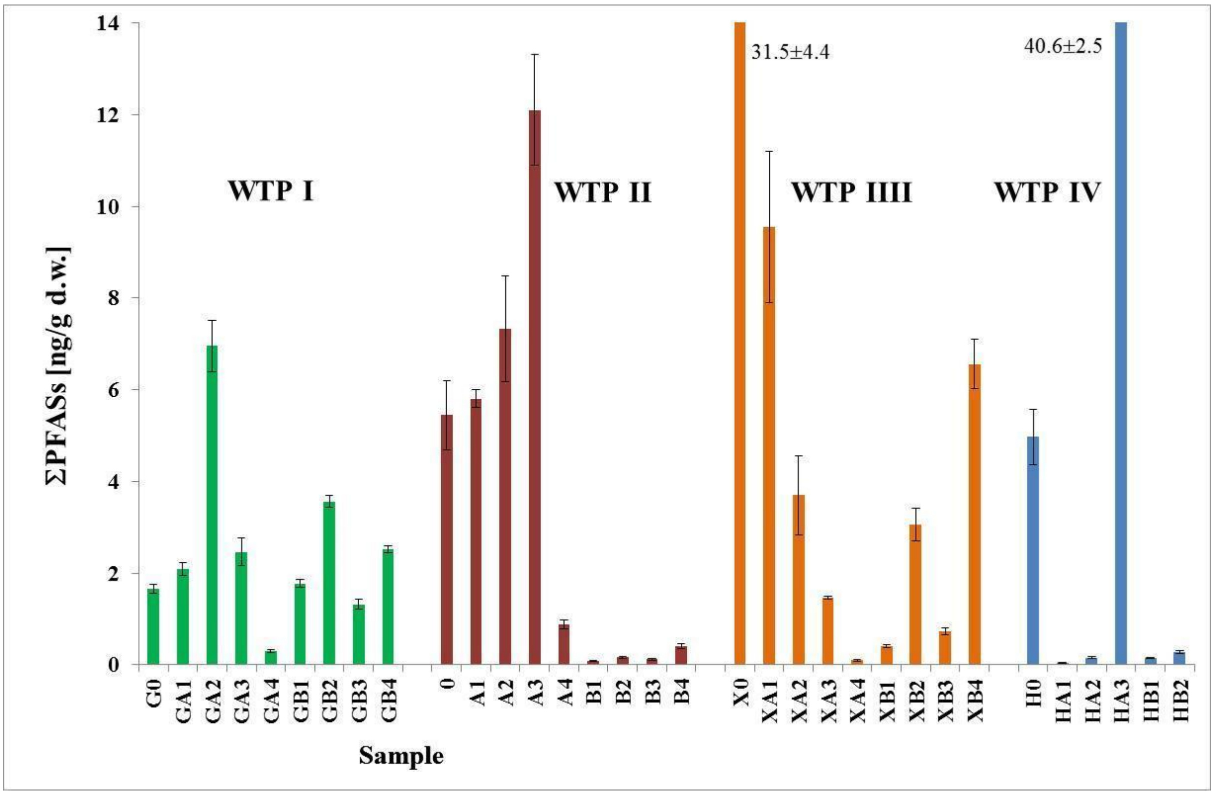


(a)

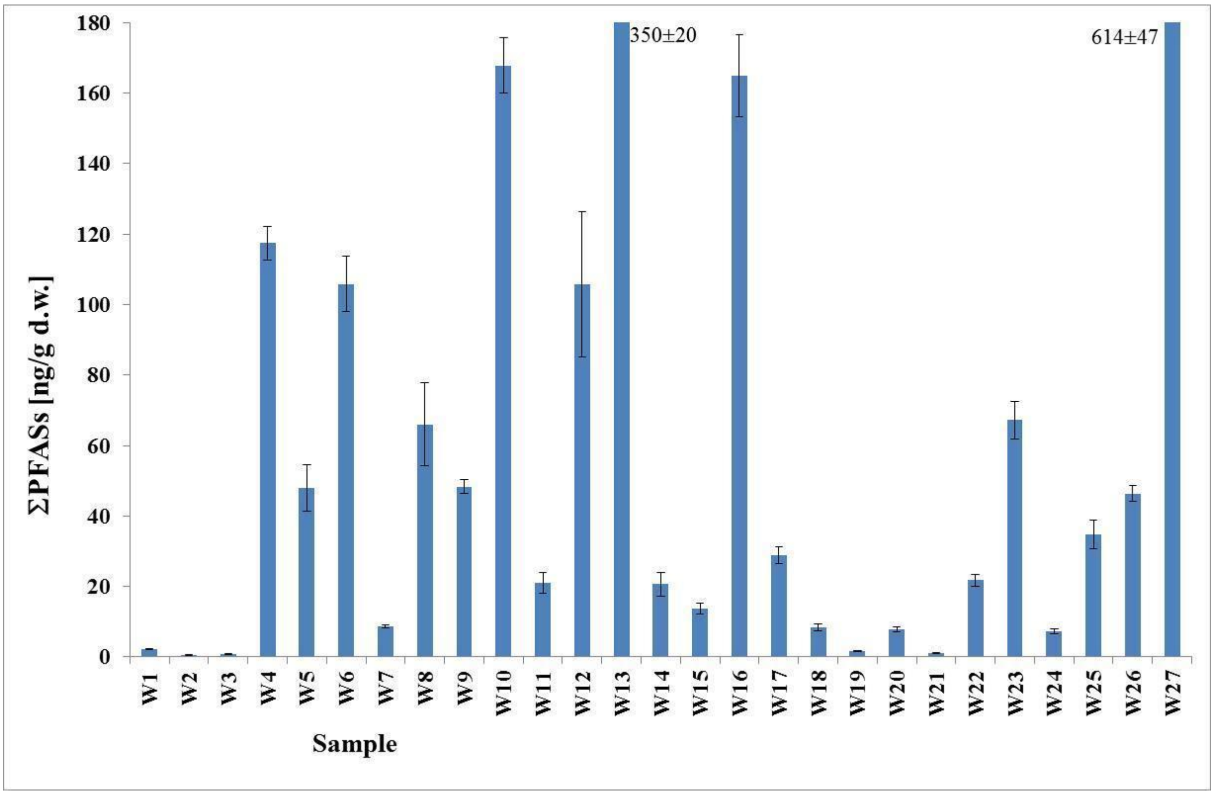


(b)

**Figure S2.** The results of the determination of PFASs in samples taken around the sewage collectors' outlets from wastewater treatment plants (WTP I-WTP IV) (a) and from the mouth of Vistula (b)
